# The stem cell factor SALL4 is an essential transcriptional regulator in mixed lineage leukemia-rearranged leukemogenesis

**DOI:** 10.1186/s13045-017-0531-y

**Published:** 2017-10-03

**Authors:** Lina Yang, Li Liu, Hong Gao, Jaya Pratap Pinnamaneni, Deepthi Sanagasetti, Vivek P. Singh, Kai Wang, Megumi Mathison, Qianzi Zhang, Fengju Chen, Qianxing Mo, Todd Rosengart, Jianchang Yang

**Affiliations:** 1 Department of Surgery and Medicine, Baylor College of Medicine (BCM), Houston, TX 77030 USA; 2 Department of Pathology, Stony Brook University Medicine, Stony Brook, NY USA; 3 Department of Medicine, Baylor College of Medicine, Houston, TX USA; 4 Dan L Duncan Cancer Center, Baylor College of Medicine, Houston, TX USA

**Keywords:** Transcription factor, Epigenetic, DOT1l, LSD1, Histone methylation, Hematopoietic stem cells

## Abstract

**Background:**

The stem cell factor spalt-like transcription factor 4 (SALL4) plays important roles in normal hematopoiesis and also in leukemogenesis. We previously reported that SALL4 exerts its effect by recruiting important epigenetic factors such as DNA methyltransferases DNMT1 and lysine-specific demethylase 1 (LSD1/KDM1A). Both of these proteins are critically involved in mixed lineage leukemia (MLL)-rearranged (MLL-r) leukemia, which has a very poor clinical prognosis. Recently, SALL4 has been further linked to the functions of MLL and its target gene homeobox A9 (HOXA9). However, it remains unclear whether SALL4 is indeed a key player in MLL-r leukemia pathogenesis.

**Methods:**

Using a mouse bone marrow retroviral transduction/ transplantation approach combined with tamoxifen-inducible, CreER^T2^-mediated *Sall4* gene deletion, we studied SALL4 functions in leukemic transformation that was induced by MLL-AF9—one of the most common MLL-r oncoproteins found in patients. In addition, the underlying transcriptional and epigenetic mechanisms were explored using chromatin immunoprecipitation (ChIP) sequencing (ChIP-Seq), mRNA microarray, qRT-PCR, histone modification, co-immunoprecipitation (co-IP), cell cycle, and apoptosis assays. The effects of SALL4 loss on normal hematopoiesis in mice were also investigated.

**Results:**

In vitro and in vivo studies revealed that SALL4 expression is critically required for MLL-AF9-induced leukemic transformation and disease progression in mice. Loss of SALL4 in MLL-AF9-transformed cells induced apoptosis and cell cycle arrest at G1. ChIP-Seq assay identified that Sall4 binds to key MLL-AF9 target genes and important MLL-r or non-MLL-r leukemia-related genes. ChIP-PCR assays indicated that SALL4 affects the levels of the histone modification markers H3K79me2/3 and H3K4me3 at MLL-AF9 target gene promoters by physically interacting with DOT1-like histone H3K79 methyltransferase (DOT1l) and LSD1/KDM1A, and thereby regulates transcript expression. Surprisingly, normal *Sall4* ^*f/f*^/CreER^T2^ mice treated with tamoxifen or *vav*-Cre-mediated (hematopoietic-specific) *Sall4* ^−/−^ mice were healthy and displayed no significant hematopoietic defects.

**Conclusions:**

Our findings indicate that SALL4 critically contributes to MLL-AF9-induced leukemia, unraveling the underlying transcriptional and epigenetic mechanisms in this disease and suggesting that selectively targeting the SALL4 pathway may be a promising approach for managing human MLL-r leukemia.

**Electronic supplementary material:**

The online version of this article (10.1186/s13045-017-0531-y) contains supplementary material, which is available to authorized users.

## Background

SALL4 is a zinc-finger transcription factor essential for developmental events and embryonic stem cell (ESC) property maintenance [1, 2]. It regulates cell type-specific gene expression programs by interacting with OCT4, SOX2, NANOG, and other “core” pluripotency transcription factors [3–6]. SALL4 is also a potent tissue stem cell factor. In normal bone marrows (BMs), it is highly expressed in hematopoietic stem/progenitor cells (HSPCs) but decreased in mature blood elements. In cultured HSPCs, forced overexpression of SALL4 markedly upregulated important HSC genes *Meis1*, *Cd34*, *Runx1*, *Bmi1*, *cMyc*, cyclins, and HOX factors, which led to prolonged ex vivo cell expansion and enhanced cell repopulating in vivo [7–9].

*SALL4* could be one of a few genes that bridge the unique properties of stem cells and malignancies. Although downregulated or absent in most adult tissues, abnormal SALL4 expression has been detected in various human tumors and leukemias which include acute myeloid leukemia (AML), B-acute lymphoblastic leukemia, and chronic myeloid leukemia (for a review, see Ref. [10]). Moreover, SALL4 expression was enriched in the side population (SP) of tumor cells, implicating its roles in cancer initiation and drug resistance [11]. In human AMLs, SALL4 knockdown caused massive cellular apoptosis and great cell growth arrest [12], while overexpression of SALL4 largely blocked myeloid differentiation and apoptosis that was induced by all-trans retinoic acid (ATRA) [13]. In animal studies, transgenic mice overexpressing SALL4 (the -B isoform) developed myelodysplastic syndrome (MDS) and AML features, and their BM HSPCs displayed increased serial replating potential [14] which rapidly induced leukemia in secondarily transplanted mice, indicating the presence of leukemia-initiating cells (LICs).

It is becoming clear that the SALL4 regulatory functions are associated with a variety of chromatin-modifying factors which include DNA methyltransferases (DNMT-1, DNMT-3A, DNMT-3B, DNMT-3L) [15], the nucleosome remodeling and deacetylase (NuRD) complex components HDAC1 /HDAC2 [16], the histone demethylase LSD1/ KDM1A [17], and others [10]. SALL4 appears to selectively recruit these “epi-factors” to define target genes that control hematopoietic self-renewal, differentiation, and apoptosis, and thus affect their expression levels and control proper cell growth. For example, in NB4 AML cells transduced with lentiviral-SALL4 [15], there was an overall increased percentage of DNA methylation at various CpG sites of the tumor suppression gene *PTEN* promoter and *SALL4* promoter itself. In cultured mouse Lin-Sca-1+ c-kit + (LSK) HSPCs, lentiviral SALL4 overexpression or Cre-induced *Sall4* gene deletion significantly affected LSD1 binding and drastically altered H3K4me3 levels at promoters of differentiation genes *Ebf1*, *Gata1*, and tumor necrosis factor *Tnf*, which significantly altered their transcript levels [17]. In 32D myeloid progenitor cells with SALL4 overexpression, the H3K4me3 and H3K79me2/3 levels at the SALL4-binding regions of the polycomb group gene *Bmi1* promoter were substantially increased [18]. The SALL4-mediated H3K4me3 modification is likely due to the SALL4-mixed lineage leukemia (MLL) interaction, which also induced increased H3K4me3 and H3K79me3 at *HOXA9* promoter [19]. In a separate functional study, a SALL4-specific 12-amino acid peptide interfering its interaction with epi-factors (such as HDAC1/2) induced leukemia death but caused no cytotoxic effects in normal HSPCs in culture nor impaired in vivo engraftment [20].

Recently, the SALL4 functions have been further linked with the MLL/HOXA9 pathway. SALL4 was demonstrated to interact with MLL protein, and the two factors occupy the same *HOXA9* promoter regions in hematopoietic cells [19]. Of note, MLL-fusion proteins (MFPs) caused by frequent chromatin rearrangements are potent inducers of oncogenic transformation, and their expression has been considered the main oncogenic driving force in ∼ 10% of human AML patients [21]. Remarkably, MLL-r leukemias display constant genomic stability, with very few gains or losses of chromosomal regions, but rely heavily on epigenetic dysregulation. In murine MLL-AF9—one of the most common MFPs with poor outcomes—AML model studies, depletion of either DNMT1 [22], KDM1A/LSD1 [23], or DOT1L [24–26] severely impaired leukemic transformation and disrupted disease progression.

Despite the accumulation of these findings, whether/or how SALL4 is involved in MLL-r leukemogenesis remains undetermined. In the present study, we investigated these issues and also examined the effects of SALL4 loss on normal hematopoiesis in mice, given the consideration of developing SALL4-based therapeutic strategies in the future.

## Methods

### Plasmids

The pMIG-MLL-AF9-GFP plasmid and the *ψ*-eco packaging vector were obtained from Dr. Scott Armstrong [27]. MEIS1 and HOXA9 retroviral vectors were purchased from Addgene (#21013 and #8515). The pCDH-CMV-3xFLAG-DOT1L vector was created in Baylor College of Medicine Genetic Core. All the plasmids were validated by DNA sequencing and or Western blotting (for 3xFLAG-DOT1L) or GFP fluorescence microscopy (for MLL-AF9-GFP). Detailed data are listed in Additional file 1.

### Mice and in vivo tamoxifen administration

The *Sall4* ^*flox/flox*^ mice [17, 28] have been crossed with *RosaCreER* ^*T2*^ mice (Jackson Laboratory) to generate *Sall4* ^*f*/*f*^ */CreER* ^*T2*^ mice. For in vivo Cre-recombination, tamoxifen (Sigma-Aldrich) was administered via intraperitoneal injection every 2 days (100 μL of 10 mg/mL in corn oil) for totally five times. Primers used for genotyping are the following: wild type forward: cctcccggaattgcttatct, neo reverse: ctgtccatctgcacgagact, and flox-Sall4 Cre check: gcttctgcctctggtattgc [28]. All animal experiments were approved by the Institutional Animal Care and Use Committee at Baylor College of Medicine or Stony Brook University Medicine.

### Colony-forming unit (CFU), replating, and BM transplantation

Lin-BM cells were isolated and transduced with recombinant MLL-AF9 using published protocols [17, 29]. Cells (0.5 × 10^4^) were plated in methylcellulose media (MethoCult™ M3234, STEMCELL Technologies), and replating was performed every 7–10 days. CFU (with > 50 cells) was scored during each round of plating. For subsequent cell culture, individual colonies were plucked using a P200 micropipettor and transferred to microcentrifuge tubes containing 500 μl of PBS. Pelleted cells were then resuspended in BM culture media [17] and maintained in a humid 37 °C 5%CO_2_-incubator. For in vitro recombination, 4-OHT (Sigma-Aldrich) was resuspended in ethanol and added to cell culture at final concentration of 250 nM. Media was changed daily during culture. For in vivo transplantation, MLL-AF9-transduced cells (5 × 10^5^) were injected through a tail vein into lethally irradiated (900 cGY) mice. Recipient mice were maintained on antibiotics for 2 weeks.

### Cell cycle analysis

Cells (1 × 10^6^/mL) were washed with ice-cold PBS-EDTA, centrifuged at 500*g* for 5 min, and fixed with 2 mL 70% ice-cold ethanol at 4 °C for overnight. After fixation, cells were washed again, resuspended in 500 μL PBS solution containing 1× RNAse and propidium iodide(Abcam), and then incubated at 37 °C for 30 min. Fluorescence was measured with a LSR II flow cytometer. The assay was carried out in triplicate, and 10,000 events were analyzed per experiment using the BDFACSDiva software. Data was analyzed with ModFit LT 5.0.

### BM hematopoietic stem and progenitor cell analysis

Propidium iodide (Molecular Probes) was used to exclude dead cells. Fluorescence-activated cell sorting (FACS) scheme used to isolate primary mouse BM cells are listed as follows [30]. The HSC and multipotent progenitor cells (MPP) were defined as HSC (Linneg Sca-1+ c-Kit+, LSK, CD34-CD135-CD150+ CD48−), MPP1 (LSK CD34+ CD135− CD150+ CD48−), MPP2 (LSK CD34+ CD135− CD150+ CD48+), MPP3 (LSK CD34+ CD135− CD150− CD48+), and MPP4 (LSK CD34+ CD135+ CD150− CD48+). For early hematopoietic precursors [31], BM cells were co-stained with antibodies against lineage markers, Sca1, c-kit, CD34, FcgRII/III, IL7Rα, and flk2.

### Chromatin immunoprecipitation (ChIP)

ChIP assays were conducted using a One-Step ChIP kit (Epigentek) with ChIP-grade antibodies against FLAG (Sigma), H3K4me3, H3K79me2/3 (Abcam), and LSD1 (Cell Signaling). Chromatin enrichment was evaluated by qPCR relative to IgG-pulled input DNA. The sequences of the PCR primers were described previously [25, 26].

### Microarray analysis and gene-expression analysis

Total RNA was extracted using TRIZOL reagent and purification with QIAGEN RNeasy Mini Kit. Microarray analysis was performed using Agilent SurePrint mouse G3 Exon 4x180K. The arrays were hybridized and scanned, and data was extracted using Agilent Feature Extraction Software Version 11.0.1.1. The Bioconductor “limma” package (http://bioconductor.org) was used to analyze the microarray data. The background-corrected data were log2 transformed and quantile normalized. Moderated *t* statistics were used to test if genes were differentially expressed between *Sall4* KO and control groups. Benjamini-Hochberg method was used to estimate false discovery rate (FDR). FDR < 0.05 were considered statistically significant.

### ChIP-Seq

This was performed by Active Motif (Carlsbad, CA). Briefly, 10 × 10^6^ cells were fixed with 1% formaldehyde and quenched with 0.125 M glycine. Chromatin was prepared and sonicated, and the DNA sheared to an average length of 300–500 bp. Genomic DNA (input) was prepared by treating aliquots of chromatin with RNase, proteinase K, and heat for de-crosslinking, followed by ethanol precipitation. An aliquot of chromatin (30 μg) was precleared with protein A agarose beads (Life Technologies). Genomic DNA regions of interest were isolated using 12 μg of anti-SALL4 antibody (Abcam #ab29112). The obtained complexes were eluted from the beads with SDS buffer and subjected to RNase and proteinase K treatment. Crosslinks were reversed by incubation overnight at 65 °C, and ChIP DNA was purified by phenol–chloroform extraction and ethanol precipitation. Illumina sequencing libraries were prepared from the ChIP and input DNAs by the standard consecutive enzymatic steps of end-polishing, dA-addition, and adaptor ligation. After a final PCR amplification step, the resulting DNA libraries were quantified and sequenced on NextSeq 500 (75 nt reads, single end). Reads were aligned to the mouse genome (mm10) using the BWA algorithm. Duplicate reads were removed and only uniquely mapped reads (mapping quality ≥ 25) were used for further analysis. Alignments were extended in silico at their 3′-ends to a length of 200 bp, which is the average genomic fragment length in the size-selected library, and assigned to 32-nt bins along the genome. Peak locations were determined using the MACS algorithm (v1.4.2) with a cutoff of *p* value = 1e−7. Peaks that were on the ENCODE blacklist of known false ChIP-Seq peaks were removed. Signal maps and peak locations were used as input data to the Active Motifs proprietary analysis program. Quantitative PCR (qPCR) reactions were carried out in triplicate using SYBR Green Supermix (Bio-Rad) on a CFX Connect™ Real Time PCR system. The resulting signals were normalized for primer efficiency by carrying out qPCR for each primer pair using input DNA. Primer sequences are available upon request.

### Statistical analysis

Log-rank test was used to detect difference in animal survival. Independent two-sample *t* test was used to detect difference between groups when the assumption of normality met. Otherwise, non-parametric Wilcoxon rank-sum test was used.

## Results

### Loss of SALL4 disrupted MLL-AF9-mediated transformation in vitro

**Fig. 1:**
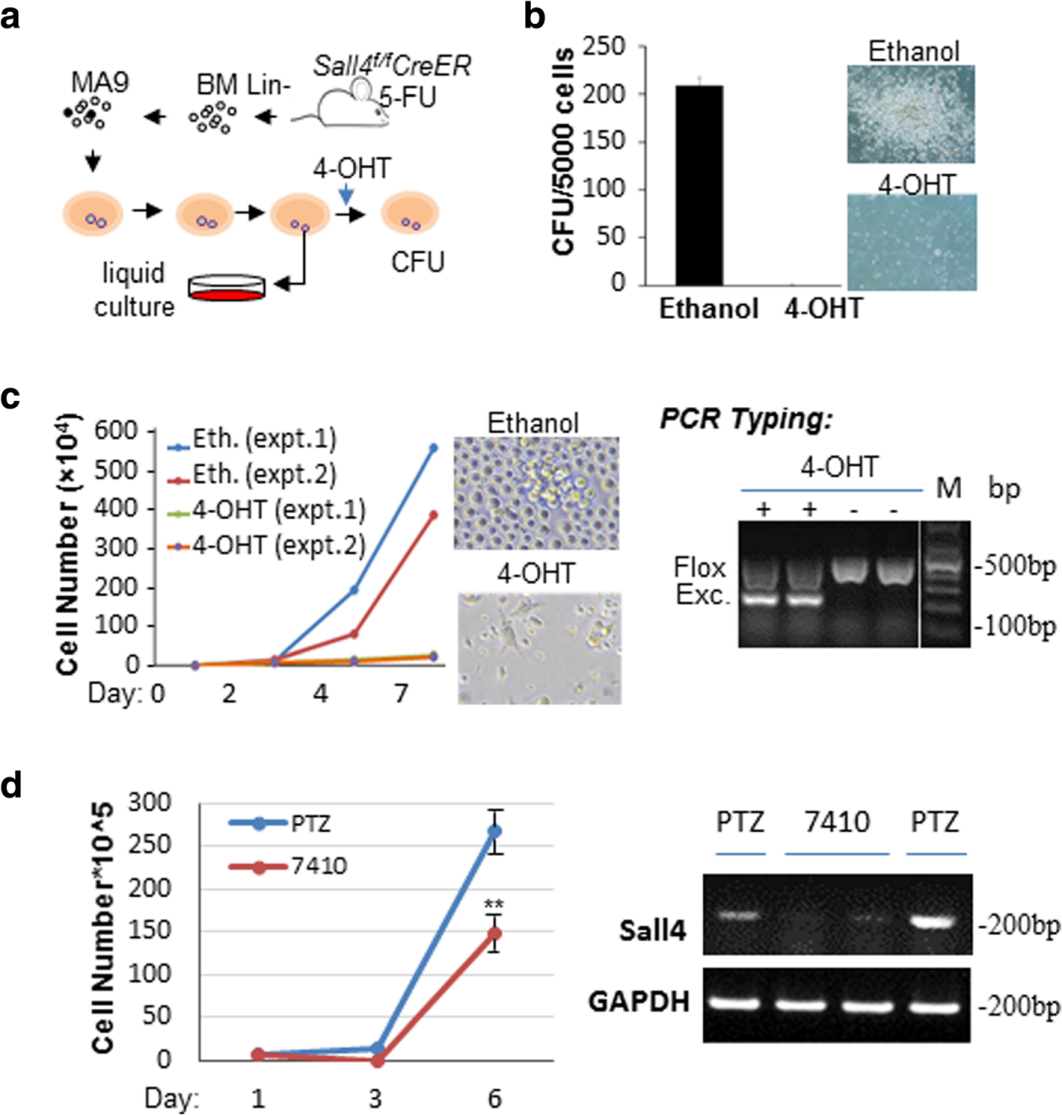
SALL4 is required for MLL-AF9-induced transformation in vitro. **a** Diagram of the in vitro procedures. **b** Relative CFU of MA9-transduced cells after ethanol or 4-OHT treatment during the fourth round plating are shown. An equal number of MA9-transformed cells were plated at each round. Representative colony images (×10) were captured using a Ti-S inverted phase/fluorescent microscope with an SPOT cooled 2.0-megapixel digital microscope camera system (Nikon, Tokyo, Japan). **c** Cells plucked from the third plating colonies were cultured in liquid and counted daily after ethanol or 4-OHT treatment. Proliferating cells were counted from two independent experiments. Representative culture images were shown (×20). The efficiency of 4-OHT-induced Sall4 excision was confirmed by PCR with primers as described previously [28]. M: DNA size marker. **d** PTRIPZ#7410 or control PTRIPZ (PTZ) vector infected MA9 cells were treated with doxycycline and viable cell numbers were counted at different days. Representative RT-PCR analysis of shRNA-induced Sall4 knockdown was shown using specific primers for mouse Sall4 or GAPDH (internal control) as described [12]. Error bars represent SD of three independent experiments. **p < 0.01

To characterize the effect of SALL4 loss on MLL-AF9-induced transformation, we first generated an inducible knockout system by crossing *Sall4-*floxed mice with mice harboring a Cre-estrogen-receptor-T2 (Cre-ER^T2^) allele at the ubiquitous *ROSA26* locus. Previous studies demonstrated that Cre-recombination of the *Sall4* allele removes exons two and three, which contain all zinc-finger domains found in *Sall4* [28]. Thus, upon tamoxifen administration, the Cre-ER^T2^ system allows for translocation of Cre recombinase into the nucleus resulting in efficient deletion of *Sall4* exons. Initially, we isolated lineage negative (Lin-) BM cells from 5-fluorouracil-primed *Sall4* ^*f/f*^CreER^T2^ mice and transduced them with a retroviral pMSCV-IRES-MLL-AF9 vector by spinoculation on three consecutive days. The cells were then subjected to four rounds of plating in methylcellulose media, with or without 4-hydroxytamoxifen (4-OHT) administration (Fig. 1a). As illustrated in Fig. 1b, c, during the fourth round of plating, 4-OHT administration induced *Sall4* exon sequence excision, which nearly abolished the clonogenic capacity of MLL-AF9-transformed cells. This was in sharp contrast to the compact, large number of “blast-like” colonies observed in ethanol (control)-treated cells. Next, individual colonies from the control group dishes have been plucked, dissociated into single cells, and grown in BM culture media. In these cultures, 4-OHT administration likewise substantially inhibited proliferation of MLL-AF9-transformed cells (Fig. 1a, c). Thus, these studies suggest that proper expression of SALL4 is required in MLL-AF9-mediated leukemic transformation. To further validate these findings with a separate model, we used our previously validated doxycycline-inducible lenti-TRIPZ-7410 *Sall4* shRNA vector [13] and transduced it into MLL-AF9-transformed BM cells. After 5 days of puromycin (1 μg/mL) selection, the recovered cells were subjected to proliferation assays and treated with doxycycline (1 μg/ml) every 2 days for 7 days. As illustrated in Fig. 1d, doxycycline-mediated *Sall4* knockdown also markedly reduced viable cell numbers in culture.

### Conditional *Sall4* knockout prevented MLL-AF9 AML initiation and attenuated disease progression in vivo

**Fig. 2:**
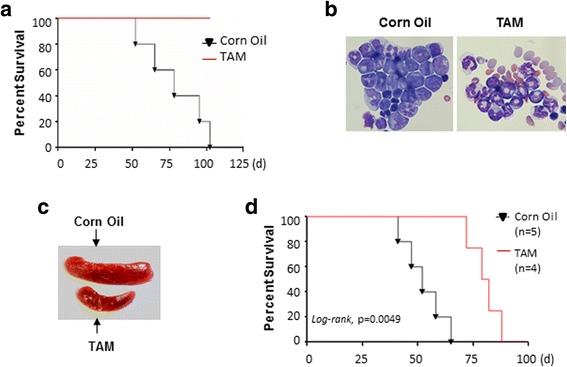
SALL4 is required for MLL-AF9-induced transformation in vivo. **a** Kaplan–Meier survival curve of mice transplanted with MA9-transduced *Sall4* ^*f/f*^ *Cre*ER^T2^ cells, with tamoxifen (TAM) or oil treatment (*n* = 5 per group). BM infiltration of AML blast cells (**b**) and enlarged spleen (**c**) are seen in oil- but not TAM-treated mice. **d** Kaplan–Meier survival curve of mice transplanted with MA9-transduced ckit+ *Sall4* ^*f/f*^ *Cre* ER^T2^ cells. TAM or oil was treated 4 weeks after cell transplantation

Based on our intriguing in vitro findings, we then sought to determine whether *Sall4* deletion also affects MLL-AF9-mediated transformation by BM transplantation—which would establish SALL4 requirement for MLL-AF9-mediated leukemogenesis in vivo. We isolated lineage Lin- BM cells from *Sall4* ^*f/f*^CreER^T2^ mice and transduced them with a retroviral MLL-AF9 vector and then injected the cells intravenously into lethally irradiated syngeneic mice. Four days later, the mice were injected intraperitoneally with either corn oil (control) or tamoxifen for 10 days to activate Cre-mediated *Sall4* excision. The mice were monitored for disease development by circulating blood cell (CBC) analysis and symptoms such as abnormal gait and labored breathing. Interestingly, while tamoxifen-administered mice failed to develop leukemia over an 8-month observation period, oil (control)-administered mice all died of AML within 102 days, as evidenced by highly elevated white blood cell (WBC) counts (not shown), marked splenomegaly, and extensive blast infiltration of BMs (Fig. 2a–c). Thus, SALL4 is clearly required for MLL-AF9-induced leukemia initiation in vivo. Next, we further asked if *Sall4* deletion at a later time point similarly affects MLL-AF9 leukemogenesis. We then generated the same MLL-AF9 leukemogenesis model, and the transplanted recipient mice were allowed to establish leukemia for up to 4 weeks. At this stage, injection of tamoxifen was found to significantly prolong the survival of the mice to a median of 80 days, compared with the median survival of 52 days of the control mice (Fig. 2d), although all mice in both groups eventually died of leukemia. These data indicate that inactivation of SALL4 is sufficient to delay MLL-AF9-induced AML progression.

### Loss of SALL4 in MLL-AF9-transformed cells induced apoptosis and cell cycle arrest at G1

**Fig. 3:**
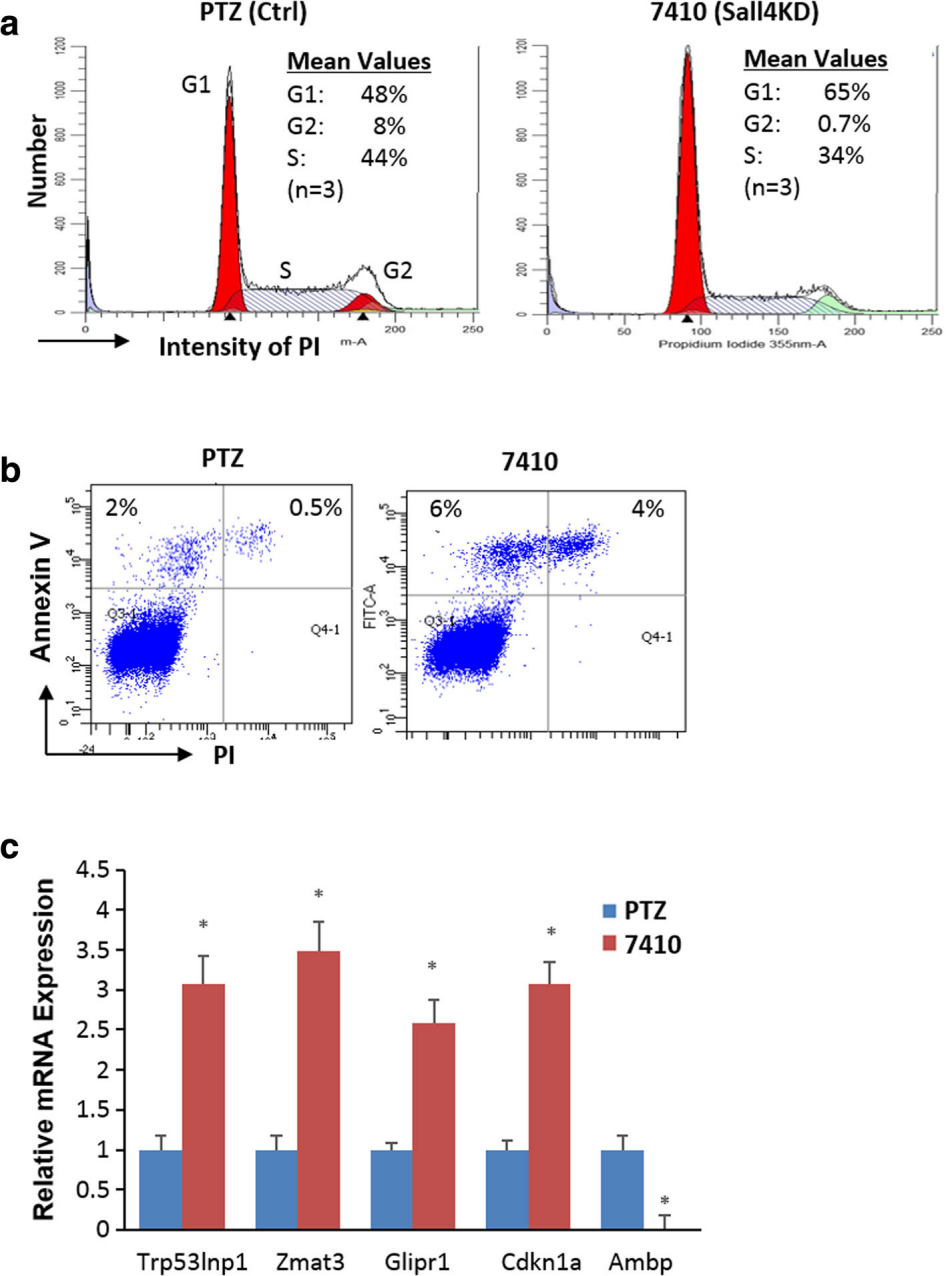
Loss of SALL4 caused apoptosis and cell cycle arrest in G1 phase in MLL-AF9-transformed cells. **a** PTRIPZ#7410 or PTZ vector infected MA9 cells were treated with doxycycline, stained with propidium iodide (PI), and their DNA content analyzed using flow cytometry. **b** Representative flow cytometry data showing Annexin V and PI stained apoptotic cells for indicated cells. **c** qRT-PCR analysis shows mRNA expression levels for indicated genes. Error bars represent SD of three independent experiments. *p < 0.05

The apparent SALL4 requirement for MLL-AF9-mediated transformation both in vitro and in vivo prompted us to test how loss of SALL4 affects MLL-AF9 cellular properties. As described in Fig. 1, 4-OHT or doxycycline treatment in MLL-AF9-transformed cells largely reduced colony-forming units and/or viable cell numbers. We next designed flow cytometric assays for cells that underwent SALL4 knockdown. We have focused on cell cycle and apoptosis markers as these have been related to SALL4 functions in AML cells [12]. As shown in Fig. 3a, propidium iodide (PI) staining and fluorescence-activated cell sorting (FACS) analyses indicate that doxycycline-mediated SALL4KD caused cell cycle arrest in cells at G1 phase (65% ± 4.8 for TRIPZ7410 vs 48% ± 2.1 for TRIPZ non-silencing vector-treated control groups, *p* = 0.005), but significantly reduced G2-phase cell fractions (0.7% ± 0.7 for TRIPZ7410 vs 7.8% ± 0.4 for control groups) and the S-phase cell populations (34% ± 3.8 for TRIPZ7410 vs 44% ± 1.7 for control groups, *p* = 0.02). In addition, PI and annexin V staining revealed that approximately 10% of the SALL4 reduction cells were apoptotic (Fig. 3b), whereas only ~ 2.5% of the control cells were apoptotic (more than a fourfold change). Consistent with the FACS data, real-time quantitative qRT-PCR analysis following SALL4 reduction showed a substantial upregulation of the transcript levels of *Cdkn1a/p21*, *Trp53inp1*, *Glipr1*, and *Zmat3/ Wig1* (Fig. 3c). Cdkn1a has been known to be a potent cyclin-dependent kinase inhibitor that blocks cell cycle progression in the G1 phase [32], while Trp53inp1 and Glipr1 are p53-induced pro-apoptotic genes that also mediate apoptosis and G1 cell cycle arrest [33, 34]. Similarly, Wig1 regulates cell cycle arrest and cell death through the regulation of p53 targets FAS and 14-3-3σ mRNA levels [35]. In contrast, transcript levels of some genes that are related with leukemia prognosis/aggressiveness, such as *Ambp* and *Thbs1*, were drastically repressed. Thus, these data suggest that deletion of *Sall4* disrupts MLL-AF9 cellular proliferation mainly by inducing apoptotic events and blocking cell cycle progression in the G1 phase.

### Loss of SALL4 affected expression levels and histone methylation status of important MLL-AF9 downstream genes

**Fig. 4:**
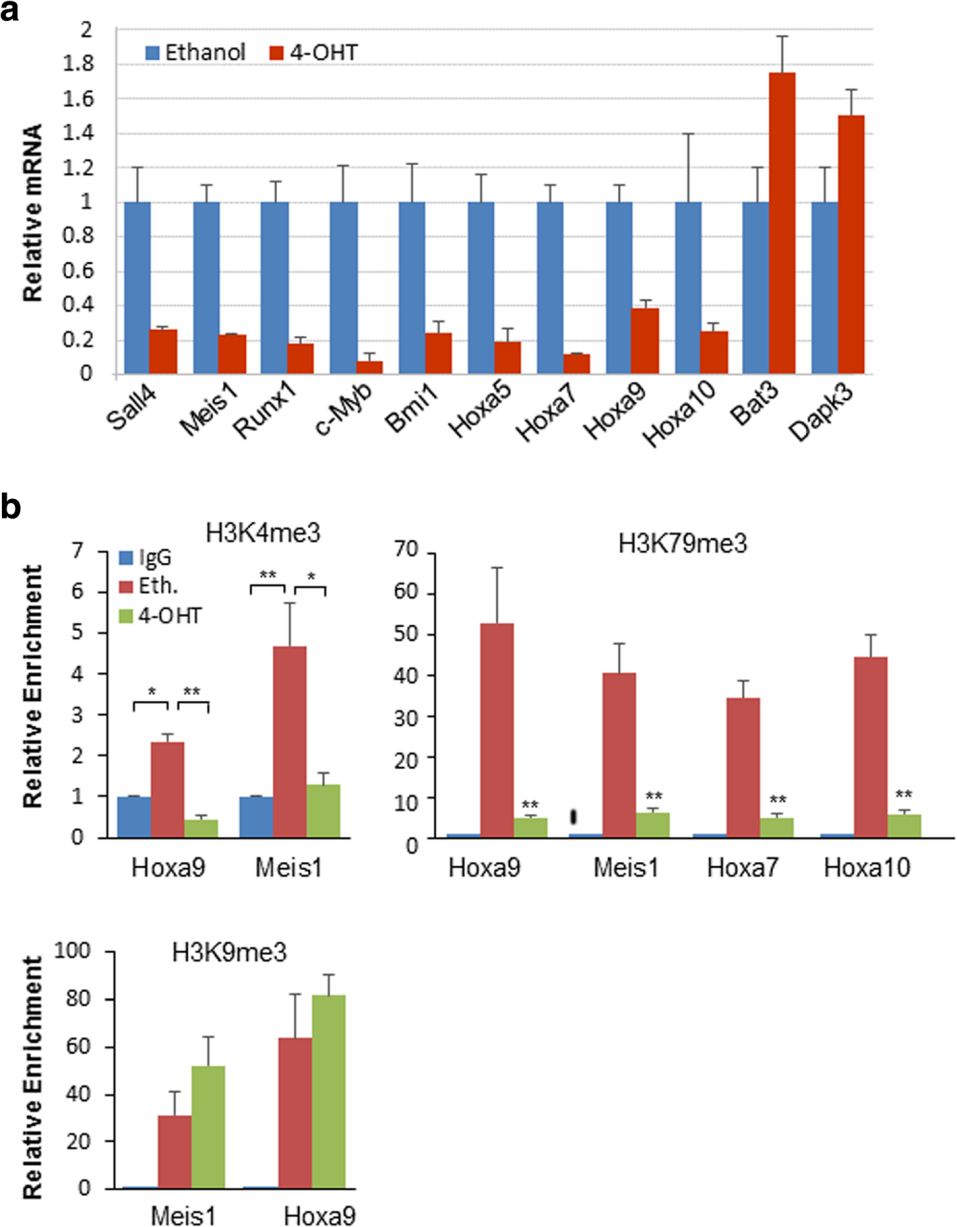
*Sall4* knockout affects expression levels and histone methylation status of important MLL-AF9 downstream genes. **a** qRT-PCR analysis shows downregulation of Meis1, HOX cluster, and multiple important MLL-AF9 target genes in cells after 5-day treatment with 4-OHT (*n* = 3). Values were normalized to GAPDH mRNA expression. **b** ChIP-qPCR assay has been conducted in MA9-transformed cells using anti-H3K79me2/3, anti-H3K4me3, and anti-H3K9me3 antibodies. Data showing that H3K79me2/3 and H3K4me3 enrichment levels at *Hoxa9*, *Meis1*, and other *Hox* gene promoter regions significantly decreased after 4-OHT treatment, which contrasts with the repressive marker H3K9me3. A 1.2-kb region upstream of the gene transcription start sites, as previously reported [18, 19, 26], were examined. Error bars represent SD of three independent experiments

In MLL-r leukemias, HOXA9 and its dimerization partner MEIS1 are the most well-characterized direct targets, which in turn can replace MLL-fusion proteins in overexpression experiments [21]. Consistent with previous findings from LSK HSPC [7] or leukemic cell [19] studies, 4-OHT-induced *Sall4* deletion in MLL-AF9-transformed BM cells decreased the transcription levels of these two factors when normalized to tested RNA levels (Fig. 4a). Additionally, multiple MLL-AF9-related genes were also downregulated, which contrasts to upregulated apoptotic genes *Bat3* and *Dapk3* (Fig. 4a). These results thus demonstrate that proper expression of MLL-AF9 target genes requires constant activation of SALL4. Next, we asked how *Sall4* deletion may affect relevant histone methylation status at the promoter regions of such genes. We focused on histone H3 lysine 4 (H3K4) and 79 (H3K79) since they have been linked with SALL4 regulatory functions and well studied in MLL-r leukemias. Indeed, SALL4 binding to *Meis1*, *Hoxa9*, and *Hox10* promoters has been determined by ChIP-Seq assays as described below. As shown in Fig. 4b, ChIP-qPCR assays clearly confirmed the enrichment of H3K4me3 and H3K79me2/3 at the proximal regions of *Meis1* and *Hox9* promoters, and 4-OHT administration in these cells fundamentally decreased their levels at all the examined sites. In contrast, while the repressive mark H3K9me3 was also found highly enriched in these promoter regions, 4-OHT administration failed to decrease their levels (Fig. 4b). Thus, in MLL-AF9-induced leukemia, SALL4 modulates MLL-AF9 downstream gene expression via defined epigenetic modification process.

### SALL4 dynamically recruited DOT1l and LSD1 to target genes in MLL-AF9-transformed cells

**Fig. 5:**
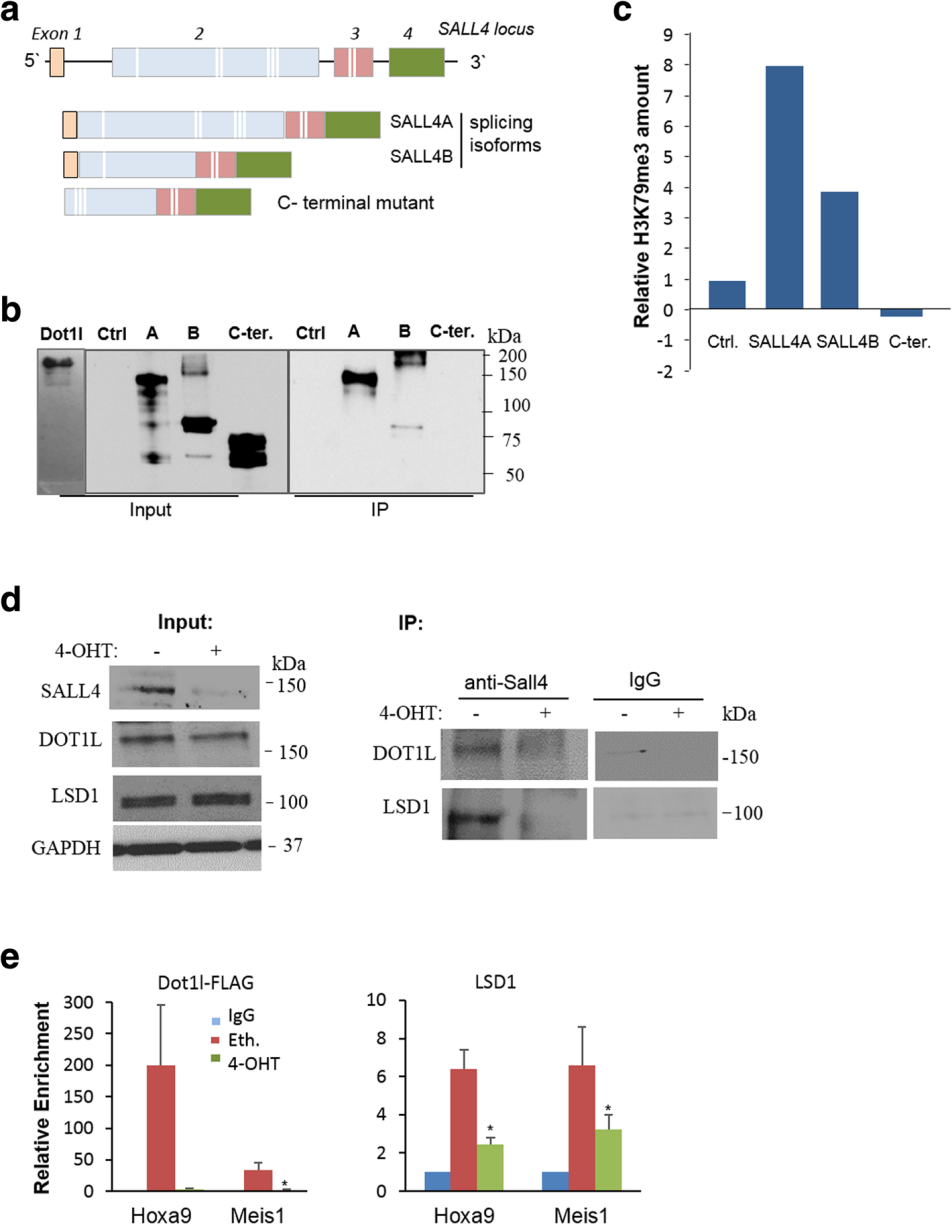
SALL4 recruits DOT1L and LSD1 to target genes in MLL-AF9-transformed cells. **a** SALL4 isoforms and C-terminal mutant are shown schematically. White lines indicate zinc-finger motifs. **b** HA-tagged SALL4 isoforms, the C-terminal mutant, and an empty vector control were transfected into 293 T cells. Their expressions and DOT1l levels are shown by Western blotting (input). An anti-DOT1L antibody (Bethyl Laboratories) was used for Dynabeads Protein G immunoprecipitation (Life Technologies). Anti-HA immunoprecipitates were analyzed by Western blotting (IP). **c** Above different SALL4-DOT1L immune-complexes were pulled down by an anti-HA antibody. The amount of tri-methylated H3K79 in the complex extracts was measured using the fluorometric EpiQuik Global Pan-methyl Histone H3K79 Quantification Kit (Epigentek) using a SpectraMax M3 microplate reader at 530–590 nm. **d** Co-IP assays were conducted with MLL-AF9-transformed Lin-BM cells, with or without 4-OHT treatment. An anti-Sall4 antibody was used for IP, and the immunoprecipitated complexes were analyzed by Western blotting using indicated antibodies (*n* ≥ 2). **e** ChIP analysis demonstrates that in MA9-transformed cells, 4-OHT treatment caused reduced binding of Dot1l and Lsd1 at *Hoxa9* and *Meis*1 promoter regions. Error bars represent SD of three independent experiments. *p < 0.05; **p < 0.01

The altered H3K4me3 enrichment at MLL-AF9 target genes should be associated with the methyltransferase activities of at least wild type MLL and LSD1, as both factors have previously been shown to interact with SALL4, while MFPs have lost H3K4 methyltransferase activity [19, 24–26]. We then considered the potential of a direct SALL4-DOT1L interaction. In previous studies, SALL4 overexpression caused enhanced H3K4me3 and H3K79me2/3 at *Bmi1* promoter in 32D myeloid progenitor cell and at *HOXA9* promoter in AML cells respectively [18, 19]. Additionally, *Hox7* and *Hox10* promoters also showed significantly decreased H3K79me2/3 following 4-OHT administration (Fig. 4b). In transfected 293 T cells, we detected that HA-tagged SALL4A and SALL4B isoforms were immunoprecipitated by DOT1l protein, while the C-terminal fragment was not (Fig. 5a, b). Consistently, in an accompanying enzymatic activity assay, the SALL4 isoforms, but not the C-terminus truncated mutant, purified increased tri-methylated H3K79 from the chromatins (Fig. 5c). We next sought to confirm their protein interactions in MLL-AF9-transformed BM cells. As shown in Fig. 5d, by a co-IP assay using an IP-grade anti-SALL4 antibody, SALL4-DOT1L interaction was clearly demonstrated by endogenous protein pulldown. Moreover, when the cells were subjected to 4-OHT-mediated *Sall4* deletion, we observed reduced DOT1L levels that were immune-precipitated by SALL4. Notably, LSD1, the previously identified SALL4-bound epigenetic factor [17] was also found immunoprecipitated by SALL4 and showed reduced pulldown after 4-OHT administration (Fig. 5d).

We then further evaluated these co-IP findings with a subsequent ChIP-qPCR assay. Because there is no available ChIP-grade anti-DOT1L antibody, we transduced the MLL-AF9 BM cells with our newly generated DOT1L-FLAG-expressing lentiviral vector (by BCM Vector Core), and its expression has been confirmed by Western blotting using antibodies against the Flag tag and DOT1L protein (not shown). As expected, both DOT1L-FLAG and LSD1 were detected bound to the *Meis1* and *Hoxa9* promoters, and 4-OHT treatment in these cells largely reduced their enrichment at the same examined promoter regions (Fig. 5e). Thus, this group data supports that SALL4 dynamically recruits DOT1L and LSD1 and regulates important downstream gene expressions in MLL-AF9 leukemic cells.

### Putative target genes of SALL4 in MLL-AF9-transformed cells

Given the clear regulatory effects of SALL4 on *Hoxa9* and *Meis1* mRNA expressions, we initially performed rescue experiments using CFU and proliferation assays. However, following 4-OHT-mediated *Sall4* deletion, combined overexpression of HOXA9 and MEIS1 (by retroviral transduction) failed to restore the MLL-AF9 transforming activity, although they could induce leukemogenesis in tested mice (not shown). Therefore, SALL4 may exert a relatively “proximal” effect in this model, or these factors control important but non-overlapping regulatory pathways. To further elucidate SALL4-regulated molecular networks, we conducted mRNA microarray assays with the MLL-AF9-transformed BM cells. We started with a relatively earlier event (where 4-OHT was treated in cells for 60 h) with the goal of detecting an “acute” effect following *Sall4* deletion. Around 77% reduction of *Sall4* mRNA levels has been verified by qRT-PCR, and the 106 up- and 57 downregulated genes were analyzed with FDR (false discovery rate) < 0.05. The complete results from the microarray analysis will be published elsewhere. Interestingly, the upregulated genes include tumor suppression/proapoptotic factors *Glipr1*, *Trpm2*, *Trp53inp1*, and *Wig1 (Zmat3)*; cell cycle inhibitors *Cdkn1a (p21)*, *Trp53inp1*, and *Wig1*; the retinoic acid (RA) biosynthesis inhibitor *Dhrs3*; HSPC colony-forming repressor *Slfn2*; and hematopoietic differentiation markers *Col5a1*, *Fyb*, *Irf8*, and *Pira*6. In contrast, the markedly downregulated genes include TGFβ factors *Tgfβ2*, *Tgfβ3*, and *Tgfβr3*, which are all critical regulators in maintaining proper proliferation of HSPCs [36, 37]. Some other downregulated genes are related to chemo-resistant or leukemia aggressiveness such as *Thbs1*, *Tgm2*, *Ambp*, and the *AF9* regulator *Sgk1*, which negatively regulates the DOT1A-AF9 repressor complex [38, 39]. Further, PANTHER (Protein ANalysis THrough Evolutionary Relationships) classification revealed that inflammation mediated by chemokine and cytokine signaling pathway (*Ccl3*, *Ccl4*) and gonadal hormone receptors (*Jun*, *Igf1*, *Dusp1*)—the newly identified leukemic cell stimulators [40]—are all affected by SALL4 levels (see Additional file 1C). These data support that SALL4 likely regulates MLL-AF9 leukemogenesis via multiple signaling pathways involving cell cycle arrest, differentiation, apoptosis induction, chemo-resistance, and importantly, HSPC, or leukemic stem cell (LSC) essential networks such as TGFβ. Further in-depth functional assays are needed to prove this notion. Το validate the microarray results, we performed RT-PCR analysis on 14 of the differentially regulated genes, which confirmed the findings. Some of the data are shown in Fig. 3c.

**Fig. 6:**
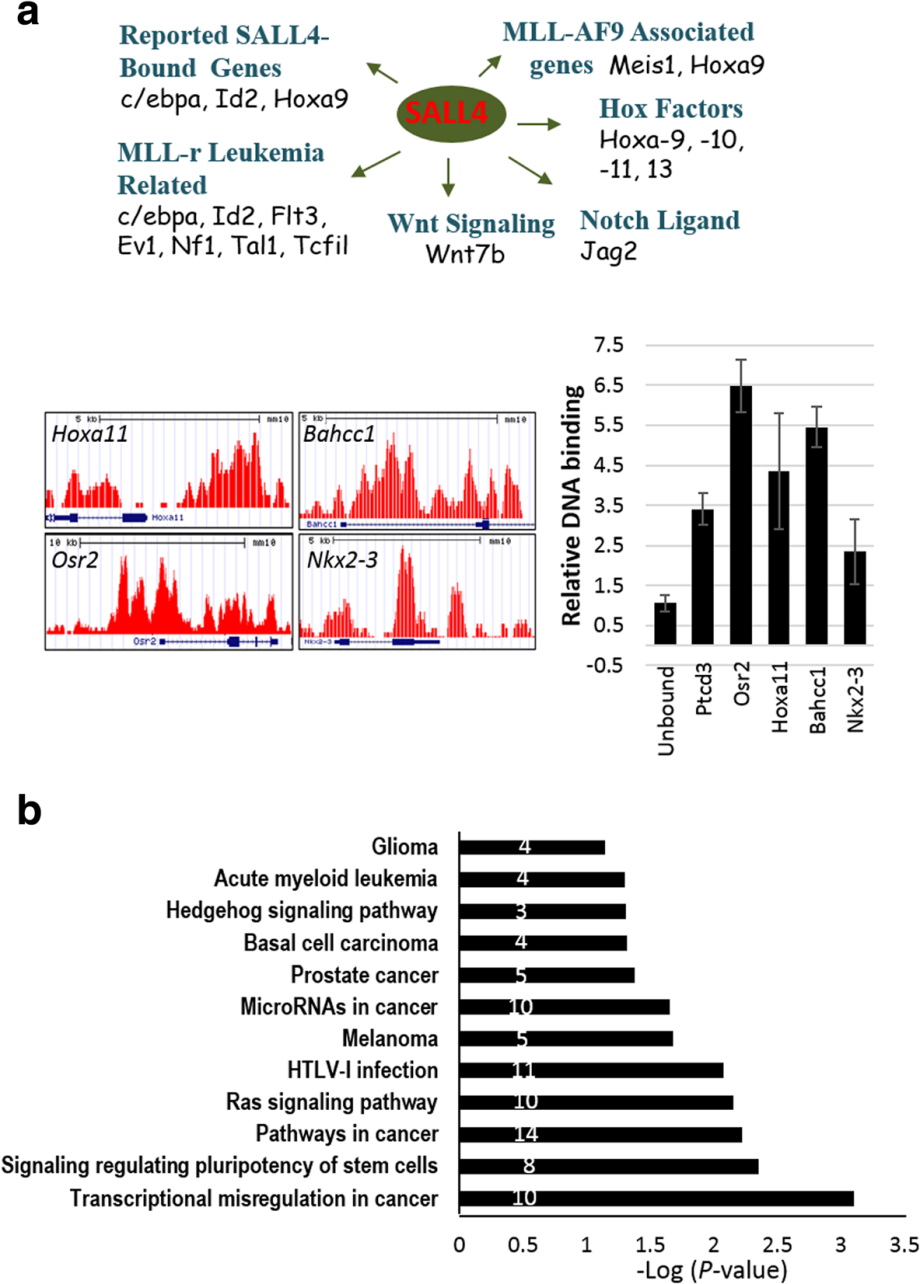
Analysis of SALL4 binding genes. **a** ChIP-Seq-identified SALL4 binding genes are classified. Representative screenshots of ChIP-Seq results displayed from the UCSC genome browser are shown. ChIP-qPCR on indicated genes is indicated as fold change over the unbound control untr17. Ptcd3 serves as a positive control locus. **b** DAVID gene ontology and KEGG_Pathway analysis of SALL4-bound genes. Involved gene numbers are shown. The detected pathways are ordered by *p* value significance (transformed by -10log10 of *p* value; highest number is the most significant). Error bars represent SD of three independent experiments

In an attempt to further classify the direct regulatory targets of SALL4, we next performed ChIP-Seq assays. High-stringency data analysis identified 350 SALL4-enriched peaks. The 350 peaks were associated with 451 protein-coding genes, including key MLL-AF9 target genes, previously identified SALL4-bound genes, and important leukemia-related genes, such as *Cebpα*, *Id2*, *Elf1*, *Evl*, *Flt3*, *Meis1*, *Nf1*, *Tal1*, *Tcf7l1*, *Gata6*, *Sox12*, *Bahcc1*, *Nkx2-3* [12, 17, 41–44], Hox factors *Hoxa9*, *Hoxa10*, *Hoxa11*, *Hoxa13*, Notch ligand *Jag2*, and Wnt/β-catenin signaling regulator *Wnt7b* (Fig. 6a and see Additional file 2 for a full list). ChIP-qPCR has been conducted on four selected binding sites, plus one positive and an unbound negative control, which confirmed the findings (Fig. 6a). Analysis of the 451 genes using the DAVID bioinformatics resources [45] identified 12 significantly enriched pathways (*p* ≤ 0.05), including cancer/AML-specific pathways, signaling pathway-regulating pluripotency of stem cells, and Hedgehog (Hh) signaling pathways (Fig. 6b). These data again support an important role of Sall4 in MLL-r leukemogenesis. In comparison with the mRNA expression data, we found that not many of the ChIP-Seq-identified SALL4 targets were associated with early expression changes as those detected in mRNA microarray assays. Considering SALL4’s dynamic and complex network in gene regulation, the limited overlap between binding and differential expression could be in part related to the length of time that 4-OHT was treated, the presence of other co-regulators in play, and/or the relatively lower number of genes that were identified in present assays. Further in-depth studies would help to fully clarify these issues.

### Deletion of *Sall4* barely affected normal hematopoiesis in mice

**Fig. 7:**
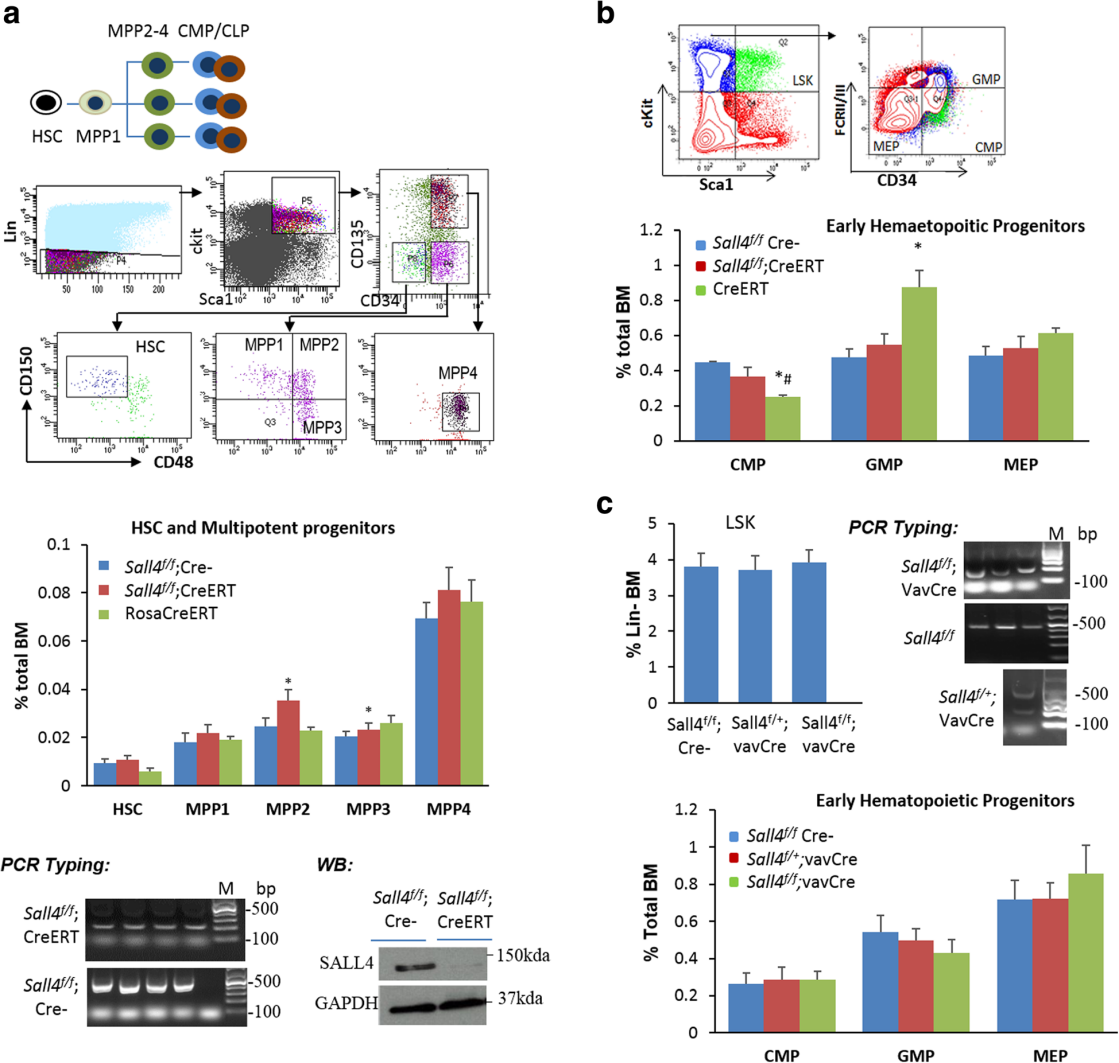
The BM stem/progenitor populations corresponding to HSC and MPP1–MPP4 (**a**) and each HPC fractions (**b**) were FACS analyzed. Representative flow cytometry scheme and quantification of the BM HSPC compartments were shown from *Sall4* ^*f/f*^, *Sall4* ^*f/f*^ CreER^T2^, and *Rosa*CreER^T2^mice 14 days after TAM treatment (*n* = 3 per group). PCR typing (4 mouse samples per group) and representative Western blotting assays for SALL4 deletion in TAM-treated *Sall4* ^*f/f*^; CreER^T2^ mice are shown. **c** Representative flow cytometry scheme and quantification of the BM HSPC compartments from *Sall4* ^*f/f*^Cre-, *Sall4* ^*f/+*^ */*vavCre, and *Sall4* ^*f/f*^/vavCre mice (*n* = 4 per group, 2–3-weeks old). PCR typing showing *Sall4* excision in indicated mouse BM cells. Error bars represent SD of at least three independent experiments. *p < 0.05 when compared to *Sall4* ^*f/f*^/CreERT; #*p* < 0.05 compared to *Sall4* ^*f/f*^Cre-. LSK Lin-Sca1 + cKit+; GMP granulocyte-macrophage progenitor; CMP common myeloid progenitor; MEP megakaryocyte-erythroid progenitor

Given our long-term goal of developing SALL4-based therapeutic strategy, we have asked how SALL4 depletion may affect normal hematopoiesis in mice. As described in Fig. 2a, mice receiving tamoxifen failed to develop leukemia, and all mice were viable without apparent phenotype defects. To gain a better understanding of SALL4 requirement in normal hematopoiesis, we then injected 8-week-old *Sall4* ^*f/f*^/CreER^T2^, *Sall4* ^*f/f*^/Cre-, and *RosaCreER* ^*T2*^ control mice with tamoxifen and verified *Sall4* excision as we did with the MLL-AF9 AML model. Consistently, in these studies, tamoxifen-mediated *Sall4* deletion did not cause significant differences in peripheral myeloid, erythroid, and platelet counts, nor BM cellularity as compared with their respective littermate controls (*Sall4* ^*f/f*^/Cre-). The *RosaCreER* ^*T2*^ control mice showed altered WBC and monocyte numbers, but they are still within the normal range (Table 1 and not shown). To examine the effect of *Sall4* deletion in each of the BM hematopoietic stem and progenitor compartments, we then performed multi-parameter flow cytometry analyses. Surprisingly, as shown in Fig. 7a–b, no significant differences in the number of HSC, multipotent progenitor MPP1, MPP4, CMP, or MEP populations were observed between *Sall4* knockout and their respective controls, and the multipotent progenitor MPP2 and MPP4 populations were even increased in TAM-treated *Sall4* ^*f/f*^/CreER^T2^ mice.

**Table 1:** CBC counts of *Sall4* ^*f/f*^ *CreER* ^*T2*^, *Sall4* ^*f/f*^ *Cre-*, and *Rosa*CreER^T2^ control animals 14 days after TAM treatment (*n* ≥ 3 for each group). Blood counts were obtained with a Hemavet950 cell counter

Group	WBC	RBC	NEUT	LYMPH	MONO	HCT
*Sall4* ^*f/f*^ *CreER*	8.3 ± 1.8	9.4 ± 0.2	9.7 ± 0.4	85.6 ± 0.4	1.3 ± 0.1	46.7 ± 1.2
*Sall4* ^*f/f*^ *Cre-*	10.0 ± 0.4	9.9 ± 0.2	10.8 ± 0.8	84.2 ± 0.7	1.3 ± 0.2	51.1 ± 1.4
*RosaCreER*	6.0 ± 0.6	9.6 ± 0.4	9.1 ± 0.6	82.8 ± 0.6	3.5 ± 0.9	50.6 ± 2.0

To further validate above in vivo findings with a separate model, we then attempted to cross *Sall4* ^*f/f*^ mice with *vav*-Cre mice (Jackson Laboratory) to delete *Sall4* throughout the hematopoietic compartment during embryonal development, even though *Sall4* homozygous knockout has previously been shown embryonic lethal [3, 28]. Interestingly, breeding of the *Sall4* ^*f/f*^/*vav*-Cre mice were successful from all of the five groups of breeder pairs that were examined, and *Sall4* ^*f/f*^/*vav*-Cre mice were born at normal Mendelian frequency with essentially normal body and organ weights. PCR typing also confirmed excision of *Sall4* exon sequences from the analyzed hematopoietic samples (Fig. 7c). Again, in these hematopoietic-specific *Sall4* deletion mice, no significant differences in basal BM hematological parameters and each HSPC fractions were detected. Thus, *Sall4* deletion causes no adverse effect on normal hematopoiesis in mice in present models (Table 2 and Fig. 7c).

**Table 2:** CBC counts of *Sall4* ^*f/f*^ *Cre-* (*n* = 6), *Sall4* ^*f/+*^ *vavCre* (*n* = 3), and *Sall4* ^*f/f*^ *vavCre* (*n* = 5) mice. The *p* values vary from 0.7 to 0.3

Group	WBC	RBC	NEUT	LYMPH	MONO	HCT
*Sall4* ^*f/f*^Cre-	4.8 ± 0.9	6.8 ± 1.0	9.7 ± 1.9	73.5 ± 7.6	2.6 ± 1.3	38.3 ± 6.2
*Sall4* ^*f/+*^vavCre	5.9 ± 0.4	7.7 ± 1.3	7.7 ± 2.3	83.1 ± 2.1	3.6 ± 0.7	45.2 ± 6.2
*Sall4* ^*f/f*^vavCre	5.3 ± 0.8	7.1 ± 0.9	8.1 ± 0.3	83.6 ± 0.7	2.6 ± 0.6	40.7 ± 4.9

## Discussion

We report here that loss of SALL4 completely inhibits MLL-AF9 AML initiation and significantly prolongs the latency of disease onset in mouse models. Importantly, SALL4 inactivation did not affect normal hematopoiesis in both conditional global gene targeting and *vav*-mediated, hematopoietic-specific gene deletion systems. These in vivo findings strongly support that SALL4 and its regulated networks as ideal therapeutic targets in treating human MLL-AF9 leukemia. Moreover, SALL4 has previously been demonstrated to physically interact with MLL at the MLL-BP domain (N-terminal), which is preserved in both wild type MLL and rearranged MFPs [19], thus SALL4 requirement may apply to a wide range of MLL-r leukemias driven by different MFPs. Clinically, MLL-r oncoproteins have been found in > 70% of infant leukemia, ~ 10% of adult AML, and many cases of secondary acute leukemias [46]. While aberrant SALL4 protein expression has been reported in most human AML cases [14], our work is thus novel in identifying therapeutic targets for this group of notorious malignancies. In future studies, it will be interesting to thoroughly characterize a potential link between SALL4 and MFP expression status in relevant leukemia patients.

In this study, we also report that SALL4 dynamically recruits the histone demethylase LSD1 and the H3K79 methyltransferase DOT1L. Recent studies demonstrated that LSD1 binding at MLL-AF9 target gene promoters decreases the H3K4me2 to H3K4me3 ratio, which promotes MLL-AF9 oncogenic gene program [23, 47]. Similarly, DOT1l can interact with AF9, and the degree of DOT1L recruitment to MLL-AF9 defines target gene H3K79 methylation levels and transformation potential [24, 26]. It would be possible that SALL4 and MLL-AF9 together orchestrate recruitment of these epigenetic modifiers, which cooperatively regulate local chromatin structure and coordinately control target gene expression, thereby modulating subsequent proper cell survival. In future studies, it will be necessary to determine if/or how SALL4 expression levels affect DOT1L binding status to MLL-AF9 or whether SALL4 expression levels substantially affect H3K4 and H4K79 methylation status at yet unidentified important MLL-AF9 target genes via these associated epi-factors.

In mRNA microarray assays, we have initially focused on a relatively earlier event with a goal of better identifying SALL4 “direct” downstream targets in MLL-AF9 leukemia. Interestingly, we identified the retinoic acid biosynthesis inhibitor DHRS3 [48], which could likely contribute to SALL4-mediated inhibitory effects in ATRA-induced AML differentiation [13]. Further biological studies will be needed to prove this assumption. We also identified that SALL4 regulates important TGFβ factors including TGFβ2, TGFβ3, and TGFβR3. SALL4 regulation of TGFβ signaling in leukemia pathogenesis has not been reported before, while the TGFβ signaling plays critical roles in HSC self-renewal, quiescence, niche regulation, and also AML and ALL leukemogenesis [36, 37]. Notably, TGFβ stimulated proliferation of myeloid-biased HSCs (My-HSCs) but inhibited lymphoid-biased HSCs (Ly-HSCs) [49]. Therefore, more in-depth studies are needed to further dissect the effects in leukemia progression that are modulated by the putative SALL4/TGFβ pathway. Additionally, an AF9 regulator SGK1 was identified. SGK1 has been shown to disrupt the assembly of the Af9/Dot1 complex [38]. Thus, studies regarding if/how SGK1 is involved in the DOT1L/MLL-AF9 protein interaction process may provide more insight into SALL4-mediated epigenetic regulations. Notably, SALL4 ChIP-Seq assay revealed that SALL4 binds to key MLL-AF9 target genes, Hox factors, and important MLL-r leukemia-related genes. Currently, another round mRNA microarray assay with a prolonged 4-OHT treatment (5~6 days in culture) samples are on the way. The expression levels of above-discussed factors, as well as the important MLL-AF9 downstream targets, will be investigated and contrasted to further establish SALL4 governed transcriptional and epigenetic mechanisms in MLL-AF9 leukemia.

In normal animal studies, we unexpectedly identified that SALL4 inactivation barely affected normal hematopoiesis in two distinct *Sall4* deletion mouse models. In a previous study, however, shRNA-mediated *SALL4* knockdown in purified human CD34+ HSPCs resulted in reduced myeloid colony formation and impaired in vivo engraftment [44]. This discrepancy could be due to the distinct models that were examined. In contrast to in vivo models, the cultured system generally lacks essential elements such as functional compensatory factors, cytokines, bypass mechanisms, optimum hormones, and nutrients but with considerably higher cell-to-cell toxic effects. Supporting this notion, mice depleted with TGFβ, the important HSC regulator which showed potent inhibitory effects on HSPC growth in vitro, also demonstrated unperturbed hematopoiesis in vivo [50, 51]. Similarly, MEIS1 and RUNX1, both are critical in MLL-AF9 leukemogenesis and in embryonic hematopoiesis but reported less so in adulthood [52]. Notably, in another SALL4-related study [19], a 12–amino acid peptide that specifically blocks SALL4-HDAC1/2 interaction resulted in impaired leukemic engraftment in vivo similar to that of *SALL4* knockdown. However, the same peptide treatment caused no cytotoxic effect of the CD34+ HSPCs in culture, nor any negative impact on in vivo engraftment. On the other hand, it needs to be noted that some genes may exert their functions only when cells encounter transplantation or replicative stress [53]. Additionally, some Vav/Cre knockout models may demonstrate severe hematopoietic defects at very late stages [54]. Thus, in future studies, serial transplantation assays, stress induction (such as 5-FU treatment), and long-term follow-up of the *Sall4* ^flox^/VavCre mice will be needed to fully clarify SALL4KO effects on normal HSC activities. The HSC-related genes, such as HOX factors, *Bmi1*, *Runx1*, the TGFβ signaling, and in vitro stem/progenitor cell expansion also need be investigated in parallel.

## Conclusions

In summary, our work identifies indispensable roles of SALL4 and its regulated epigenetic mechanisms in MLL-AF9 leukemia, and SALL4 deficiency barely affected normal hematopoiesis in in vivo knockout models. These studies would pave the way for future SALL4-targeted therapy that disrupts MLL-r leukemias, while allowing for normal stem cell activity and regeneration.

## Additional files

Additional file 1: Supplementary data. (PDF 317 kb)

Additional file 2: Full list of SALL4-bound genes. (PDF 334 kb)

## Abbreviations

AML: Acute myeloid leukemia
ATRA: All-trans retinoic acid
CBC: Circulating blood cell
ChIP-Seq: Chromatin immunoprecipitation (ChIP) sequencing
CMP: Common myeloid progenitor
Co-IP: Co-immunoprecipitation
ESC: Embryonic stem cell
GMP: Granulocyte-macrophage progenitor
HSPCs: Hematopoietic stem/progenitor cells
LSK: Lineage-Sca-1+ c-kit+
Ly-HSCs: Lymphoid-biased HSCs
MDS: Myelodysplasic syndrome
MEP: Megakaryocyte-erythroid progenitor
MFPs: MLL-fusion proteins
MLL-r: Mixed lineage leukemia (MLL)-rearranged
MPP: Multipotent progenitor cells
My-HSCs: Myeloid-biased HSCs
NuRD: Nucleosome remodeling and deacetylase
PI: Propidium iodide
qRT-PCR: Quantitative reverse transcriptase polymerase chain reaction
