# Supplementary material for: The stem cell factor SALL4 is an essential transcriptional regulator in mixed lineage leukemia-rearranged leukemogenesis
Source: J Hematol Oncol. 2017 Oct 3;10:159. doi: 10.1186/s13045-017-0531-y (PMC5627455; doi:10.1186/s13045-017-0531-y)
Supplement: Supplementary file 1 — Supplementary data. (PDF 317 kb) [file 13045_2017_531_MOESM1_ESM.pdf]

Additional File 2

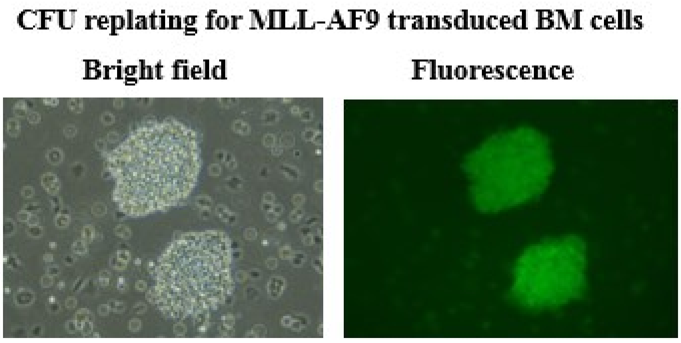

2A. MLL-AF9-GFP transformed cells were plated at each round. Representative colony images (10x) were captured using a Ti-S inverted phase/fluorescent microscope with an SPOT cooled 2.0-megapixel digital microscope camera system (Nikon, Tokyo, Japan).

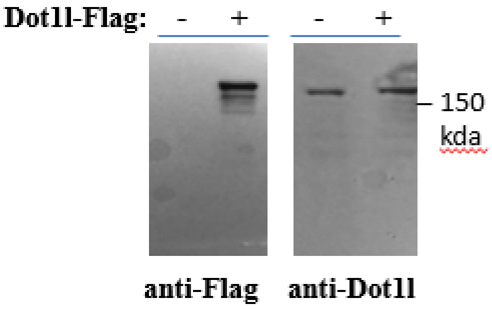

2B. Validation of 3xFLAG-DOT1L expression. Lentivirus expressing 3xFLAG-DOT1L has been pooled and transduced to proliferating cells, followed by Western blotting using antibodies specifically against the Flag tag or Dot1l protein (purchased from Sigma and Abcam respectively). +: with viral transduction; -: without transduction.

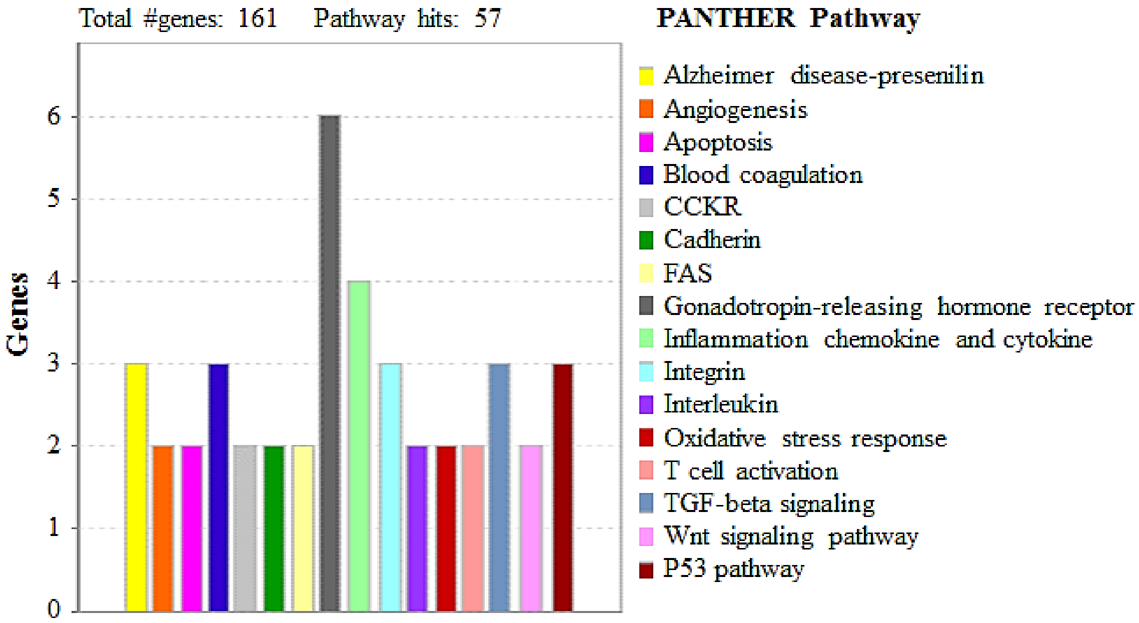

2C. PANTHER (Protein ANalysis THrough Evolutionary Relationships) classification system showing the enriched pathways that were affected by *Sall4* deletion.
