# Supplementary material for: The stem cell factor SALL4 is an essential transcriptional regulator in mixed lineage leukemia-rearranged leukemogenesis
Source: J Hematol Oncol. 2017 Oct 3;10:159. doi: 10.1186/s13045-017-0531-y (PMC5627455; doi:10.1186/s13045-017-0531-y)
Supplement: Supplementary file 2 — Full list of SALL4-bound genes. (PDF 334 kb) [file 13045_2017_531_MOESM2_ESM.pdf]

SALL4 Bound Genes

Pcdhga4
Tmed6
Slc39a10
Hecw2
Fev
Speg
Slc45a3
Pik3c2b
Cacna1s
Rnf2
Rasal2
Nos1ap
Nhlh1
Susd4
Plxna2
Hivep2
Slc35d3
Eya4
Pcdhgb2
Marcks
Amd1
Ostm1
Aim1
Grik2
D630037F22Rik
Adarb1
Nfyb
Usp44
Cnot2
Ppm1h
Tug1
Meis1
Aftph
Otx1
Tlx3
Rnf145
Maml1
Lyrm7
Pcdhga5
Kcnj12
Ulk2
Ntn1
Cpd
Nf1
Gm11423
2410003L11Rik

Lasp1
Ikzf3
Cyb561
Fam20a
Sdk2
Cdr2l
Sphk1
Jmjd6
Cbx2
Bahcc1
Nploc4
Lpin1
E2f6
Cys1
Id2
Lamb1
Ccdc71l
Foxg1
Ttc6
Trappc6b
Dnaaf2
Ccdc177
Slc8a3
Acot4
Bcl11b
Gm16596
A730018C14Rik
Jag2
Brf1
Kif13a
Pitx1
Hnrnpk
C130071C03Rik
Otp
Paip1
LOC101055764
Top2b
Txndc16
Ero1l
Ap1g2
Nefl
Wbp4
Farp1
Osr2
Tmem65
E430025E21Rik
2810039B14Rik

Dgat1
Elfn2
Pdgfb
Mcat
Wnt7b
Cerk
Spats2
Tbx1
Lpp
Fgf12
Zdhhc23
Scaf4
Gm3004
Dyrk1a
Ets2
Mdga1
Ephx3
Cul9
Ppp2r5d
1700001C19Rik
Treml2
Plcl2
St6gal2
Pja2
Tmem200c
Pcdhga6
Yipf4
Heatr5b
Arhgap12
Colec12
1010001N08Rik
Pcdhga7
Pcdhga8
Pcdhgb4
Pcdhga1
Pcdh1
Afap1l1
Pcdhga9
Skor2
Gm960
Pcdhgb7
Pcdhga10
Mzb1
2700081O15Rik
Pcdhgb6
Pcdhgb5
Foxd4

Cpeb3
Frat1
Nkx2-3
Lbx1
9130011E15Rik
Mir146b
Nt5c2
Sh3pxd2a
Emx2os
Gpr158
Lhx3
Ntng2
Gle1
Lypd6
Lrp2
Dlx1as
Sp9
Evx2
Hoxd12
Nfe2l2
Ehf
Hipk3
Grem1
H3f3c
Kcnip3
Gfra4
Sel1l2
Sox12
Pxmp4
Raly
Soga1
Fitm2
Zmynd8
Stau1
Bmp7
Dido1
Mir124a-3
Spata5
Elf2
Nhlrc3
Commd2
Shox2
She
Cgn
Igsf3
Neurog2
Ddit4l

Rap1gds1
Lrrc7
Vmn1r2
Esrp1
Tmem64
Igfbpl1
Palm2
Gng10
Snx30
Atp6v1g1
Rasef
Lrp8
Foxd2
9130206I24Rik
Tal1
Cyp4b1
Rnf220
Mycl1
Zcchc17
Ahdc1
1810019J16Rik
Arid1a
Epha8
Ece1
Pax7
Spen
6330411D24Rik
Casz1
Clstn1
Gpr153
Gatad1
Pus7
Shh
Letm1
Htra3
Lap3
1600023N17Rik
Pi4k2b
4932441J04Rik
Fam114a1
Slc30a9
Igfbp7
11-Sep
Prdm8
Fam69a
Gm10419
Fgfrl1

Sgsm1
Cabp1
Msi1
C330018A13Rik
1700048F04Rik
Wbscr22
Pdgfa
4930500L23Rik
Radil
Flt3
Mtus2
Kl
Dlx5
Mdfic
Smo
Zfp467
Fam221a
Hoxa9
Hoxa11
Scrn1
Serbp1
Tcf7l1
Dqx1
Fbxo41
Arl8b
Srgap3
Cand2
Tspan9
Vmn1r59
Npas1
Fbxo46
Atp1a3
Grik5
Sptbn4
Ttc9b
Cebpa
Shank1
Myh14
Klf13
Apba2
Igf1r
A730056A06Rik
Pde8a
Gm4980
Arhgef17
Rbmxl2
Rras2

Prr14
Mcmbp
Hmx3
Adam12
Gm5607
Gm9908
Cpe
4933436C20Rik
Ccdc79
Nae1
Vps4a
Fendrr
Sult5a1
Egln1
Tsnax
Olfm2
Ddx6
Cadm1
Neo1
Rbpms2
Ibtk
Msl2
Actl11
Smarcc1
Kif9
Trank1
Trim71
9530059O14Rik
Slc6a20a
Cryba2
2810025M15Rik
Gm20114
Pex7
5930403N24Rik
Msl3l2
5330438D12Rik
Morc2a
Ehbp1
Ranbp17
Canx
Hint1
Lig3
E130012A19Rik
B230217C12Rik
Zpbp2
Ube2o
Mettl23

Gm11772
Tspan10
3110039M20Rik
Pnn
9330151L19Rik
Acot3
Evl
B020018J22Rik
Nudt14
Mir7-1
Mir9-2
Gpr137c
Jph4
Elf1
BC048602
Ube2d4
Nsmce2
Scrt1
Tspo
4930588K23Rik
Gramd1c
Brd4
Srf
Pex6
AI661453
B430306N03Rik
Gata6
Prob1
Pcdhga2
LOC101056140
Rtn3
A330032B11Rik
2310034G01Rik
Ina
Emx2
Qsox2
6530402F18Rik
Sptan1
Dlx2
Cir1
Hoxd13
Hoxd11
E030042O20Rik
Rtf1
Adam33
Macrod2
Tldc2

2310001K24Rik
4930577N17Rik
Proser1
Rsrc1
Vmn1r3
AI481877
Slc46a2
Gm11240
9130206I24Rik
Foxe3
Snrnp40
Gm4123
Pik3cd
Hes3
4930511M11Rik
Rint1
9530036O11Rik
Med28
Ncapg
Pcdh7
4930430O22Rik
Ccdc60
Dnajc30
Uncx
Sspo
Stk31
Mir196b
Hoxa11as
Tlx2
Egr4
Edem1
Rpl32
Gm7025
Grik5
Zfp574
Map3k10
Rgma
P2ry6
Fbrs
Sec23ip
A130023I24Rik
Gm5606
Irx5
Nae1
Car7
Pdf
Foxf1

Camkv
Cspg5
Setd2
Srsf2
Pde6g
Gm9804
Pole2
Rmi1
Ptk7
Spata24
Pcdhga3
Tmem180
Hoxd12
Hoxd10
B330016D10Rik
Fam184b
Vps37d
Hoxa10
Hoxa13
Pcgf1
Snora7a
Pou2f2
Sox1
Cog8
Mfsd11
Pcdhgb1
Hoxa11
Efcab12
Nip7
